# Antiviral Immune Response in Alzheimer’s Disease: Connecting the Dots

**DOI:** 10.3389/fnins.2020.577744

**Published:** 2020-10-02

**Authors:** Ethan R. Roy, Wei Cao

**Affiliations:** Huffington Center on Aging and Department of Molecular and Human Genetics, Baylor College of Medicine, Houston, TX, United States

**Keywords:** antiviral, interferon, Alzheimer’s disease, innate immunity, amyloid, pathogen hypothesis, neuroinflammation

## Abstract

Alzheimer’s disease (AD) represents an enormous public health challenge currently and with increasing urgency in the coming decades. Our understanding of the etiology and pathogenesis of AD is rather incomplete, which is manifested in stagnated therapeutic developments. Apart from the well-established Amyloid Hypothesis of AD, gaining traction in recent years is the Pathogen Hypothesis, which postulates a causal role of infectious agents in the development of AD. Particularly, infection by viruses, among a diverse range of microorganisms, has been implicated. Recently, we described a prominent antiviral immune response in human AD brains as well as murine amyloid beta models, which has consequential effects on neuropathology. Such findings expectedly allude to the question about viral infections and AD. In this Perspective, we would like to discuss the molecular mechanism underlying the antiviral immune response, highlight how such pathway directly promotes AD pathogenesis, and depict a multilayered connection between antiviral immune response and other agents and factors relevant to AD. By tying together these threads of evidence, we provide a cohesive perspective on the uprising of antiviral immune response in AD.

## Introduction

Hallmarked by the deposition of β-amyloid plaques and accumulation of neurofibrillary tangles, Alzheimer’s disease (AD) manifests with complex pathophysiology and its etiology remains elusive. Among the many viewpoints for the underlying mechanisms, the Pathogen Hypothesis was proposed initially based on the clinical discoveries of Herpes Simplex Virus-1 (HSV-1) in association with AD, further expanded to include other microbes, and gained experimental support in recent years (Itzhaki et al., 1997, 2016; Itzhaki, 2018). In particular, Aβ has been shown to function as an antimicrobial peptide and, under experimental conditions, protect against microbial infection while seeding Aβ deposition (Kumar et al., 2016; Eimer et al., 2018). Separately, pathogenic involvement of proinflammatory responses in AD is also increasingly being appreciated (Heneka et al., 2015; Ransohoff, 2016). Because of the intimate association between infections and inflammation, the Pathogen Hypothesis putatively links to the neuroinflammation phenomenon in many aspects; however, the precise molecular correlation between these two processes in AD has yet to be established. Here, we intend to make the connections from multiple angles.

## Molecular Mechanism Underlying the Antiviral Immune Response in AD

Mammalian antiviral innate immune defense mechanism utilize an array of nucleic acid innate immune sensors to detect viral genomes or their replication products, which activate a cascade of signaling events to induce rapid gene expression (Barrat et al., 2016). Among them, the type I IFN (IFN) cytokines, which include multiple IFNα subtypes, IFNβ, IFNε IFNκ, and IFNϖ, instruct the frontline antiviral response (Capobianchi et al., 2015; McNab et al., 2015). All IFN species signal through a common receptor complex and drive the transcription of a large panel of IFN-stimulated genes (ISGs) (Schreiber and Piehler, 2015). ISGs operate in concert to interfere with viral replication through viral genome degradation and blockade of gene expression, protein synthesis and virion assembly, thus conveying protection.

Although normally non-immunogenic, host self-derived nucleic acids can provoke IFN response under pathological conditions such as autoimmune diseases. In systemic lupus erythematosus, immune complexes comprised of a patient’s DNA or RNA and associated autoantibodies activate plasmacytoid dendritic cells (pDCs), a subset of innate immune cells, to stimulate IFN production and flares of systemic inflammation (Gilliet et al., 2008). In psoriasis, antimicrobial peptide cathelicidin LL-37 complexes with nucleic acids and similarly induces aberrant IFN production from pDCs (Lande et al., 2007). In both cases, nucleic acid-containing complexes gain immunogenicity by delivering nucleic acids to the intracellular innate immune sensors in pDCs to activate the signaling cascade for IFN production.

**FIGURE 1:**
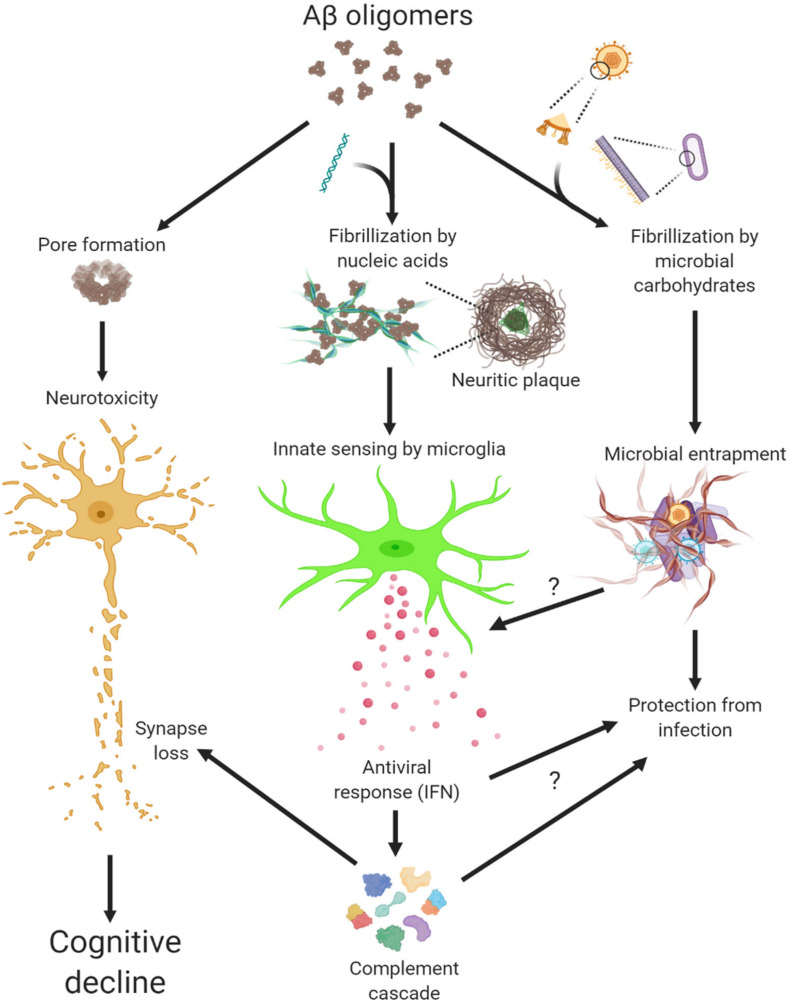
Multiple actions of Aβ oligomers leading to neuropathogenesis in Alzheimer’s disease.

LL-37 and other antimicrobial peptides oligomerize and form pore structures on biological membranes (Arnusch et al., 2007; Xhindoli et al., 2014) (Figure 1). Similarly, oligomerization of Aβ exerts neuronal toxicity as well as antimicrobial function (Kayed et al., 2003; Soscia et al., 2010). By studying oligomers made from various proteins, we not only confirmed this fascinating gain-of-function but also discovered another intrinsic property of protein oligomers – affinity toward negatively charged molecules (Di Domizio et al., 2012b). Complexing soluble oligomers with nucleic acids or glycosaminoglycan, both negatively charged, expedite the formation of amyloid fibrils *in vitro*. More strikingly, nucleic acid-containing amyloid fibrils are potent inducers of IFN response from pDCs *in vitro* and *in vivo* (Di Domizio et al., 2012a). In short, Aβ and LL-37 share several characteristics: oligomerization, cytotoxicity to host and microbe cells, and binding to cofactors, the latter conveys interferongenicity (Figure 1). Not surprisingly then, amyloid-DNA composites present in bacterial biofilm stimulate an IFN response and promote autoimmunity (Gallo et al., 2015). To immune cells, these protein-nucleic acid complexes are indiscriminately sensed as virions to trigger an antiviral immune response.

Microglia, the brain resident immune cells, fulfill important functions in trophic support, cell debris removal and tissue surveillance under homeostatic conditions (Li and Barres, 2018; Prinz et al., 2019). However, microglial dysfunction can be a primary cause to neurological disorders, thus disease-associated microglia have received intense attention in recent years. Mouse primary microglia elicited a rapid IFN response to nucleic acid-containing amyloid fibrils *in vitro*, in a manner similar to that from peripheral immune cells (Roy et al., 2020). Activated microglia expressing Clec7a not only selectively surrounded the amyloid β plaques with sequestered DNA and RNA but also upregulated a large panel of ISGs, indicating profound IFN pathway activation in mouse models of amyloidosis. Thus, IFN constitutes a pivotal element within the neuroinflammatory network of AD. Most recently, the early and persistent IFN and antiviral response was confirmed by an analysis of microglial proteomes from Aβ models (Monasor et al., 2020).

## Antiviral Immune Response in Promoting AD Pathogenesis

Microglia recruited to amyloid β plaques adopt a disease-associated phenotype, where they acquire a unique molecular state by downregulating homeostatic markers, upregulating immune effector molecules, and express a panel of genes that have been associated with increased risk for AD and other neurodegenerative disorders (Butovsky and Weiner, 2018; Hammond et al., 2018; Wang and Colonna, 2019). IFN was shown to be required for sustaining microglial activation since suppression of IFN signaling significantly reduced the levels of CD68 and Clec7a, markers for microglial activation, and shifted the morphology of microglia toward homeostatic form (Roy et al., 2020). Intriguingly, polymorphisms of ISGs, including *OAS1*, *ITGAM*, *LAPTM5*, and *LILRB4*, were conjointly identified as a significant risk factor for AD, implicating IFN pathway as a genetic modifier (Salih et al., 2019).

In AD tissue, ISG-expressing microglia preferentially associate with nucleic acid^+^ neuritic plaques (Roy et al., 2020). Furthermore, the IFN activation signature was correlated with amyloid load and disease severity across a large study cohort represented in the Mt. Sinai Brain Bank. These findings are in line with earlier reports of neuritic plaques sequestering self nucleic acids and microglia expressing IFNα in AD brains (Yamada et al., 1994; Ginsberg et al., 1997). Therefore, a prototypical antiviral immune response is manifested in both pre-clinic models and clinic AD cases. Interestingly, AD patients with rare TREM2 R47H variant, an AD risk factor, have increased presentation of proinflammatory microglia subsets including those enriched with IFN response (Sayed et al., 2020).

Although normal brain lacks detectable IFN expression, transient IFN production in CNS protects against opportunistic viral infections (Nallar and Kalvakolanu, 2014). However, chronic and dysregulated IFN expression is a major driver for type I interferonopathy, a group of hereditary CNS disorders (Hofer and Campbell, 2013; Rodero and Crow, 2016; Schwabenland et al., 2019). Separately, patients receiving systemic IFN treatment or with HIV-induced dementia display increased brain IFN activation, which is associated with cognitive and psychiatric dysfunctions (Gray et al., 1996; Hayley et al., 2013; Dipasquale et al., 2016; Wachholz et al., 2016). These observations collectively suggest a toxic influence of IFN on the brain. When delivered to the brain, IFNβ directly activates microglia, initiates neuroinflammation, and leads to microglia-mediated synapse loss (Roy et al., 2020). Conversely, suppression of IFN signaling in Aβ models rescued the synapse loss in the brain, supporting a direct role of IFN in synapse modification. Interestingly, IFN receptor ablation was previously shown to improve cognitive performance while altering glial phenotypes in APP_SWE_/PS1_Δ__E__9_ mice (Minter et al., 2016).

Complement has been implicated in synaptic pathologies in diverse neurological and neuropsychiatric diseases, in particular neurodegeneration (Hong et al., 2016; Morgan, 2018; Tenner et al., 2018; Wu et al., 2019). Intriguingly, not only do a number of complement genes represent bona fide ISGs, but IFN-stimulated synapse elimination depends on the function of complement C3 (Roy et al., 2020). Not coincidently, synapse loss and memory impairment in mice recovered from West Nile Virus (WNV) infection was shown mediated by persistent microglial activation and the functional involvement of complement C3 (Vasek et al., 2016). Moreover, IFN pathway is robustly correlated with complement cascade in human AD (Roy et al., 2020). Given that synapse loss is clinically associated with cognitive decline (DeKosky and Scheff, 1990; Scheff et al., 1990; Terry et al., 1991), these findings highlight a pathogenic role of antiviral immune response in conjunction with complement cascade in modifying synapses in AD (Figure 1).

## Contributing Factors to Antiviral Immune Response in AD

**FIGURE 2:**
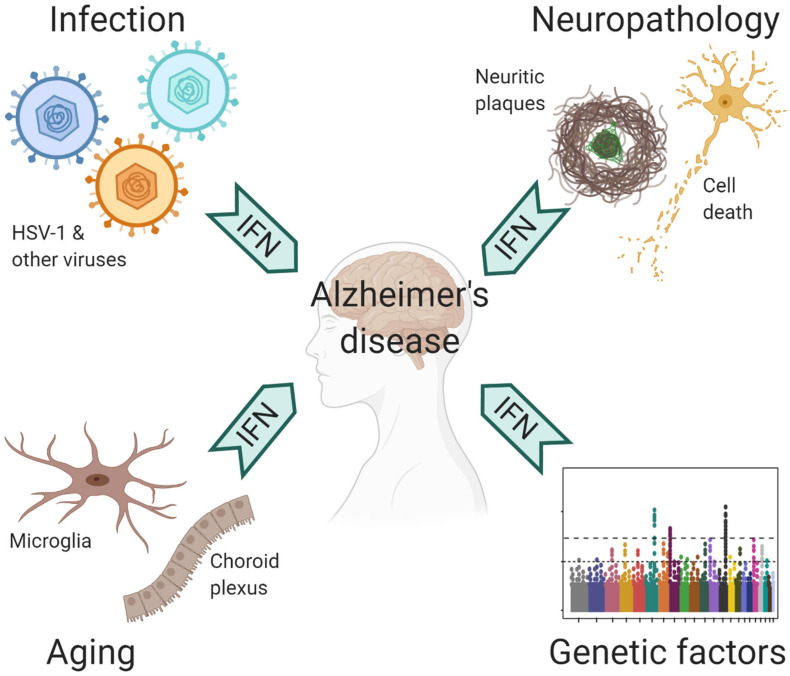
Contributing factors to antiviral immune response in AD.

The central nervous system (CNS) is protected by a highly complex barrier structure, but is by no means invincible to infections. Many neurotropic viruses can gain access to the brain through blood circulation or peripheral nerves (Swanson and McGavern, 2015). Acute viral infection in the CNS often triggers inflammatory response, where the IFN pathway plays a crucial role in the defense against a wide range of viral pathogens (Paul et al., 2007). However, strong immune reaction also leads to acute meningitis, encephalitis and myelitis, which manifest with behavioral and cognitive disruptions (Swanson and McGavern, 2015). In humans, WNV, HIV-1, Zika virus, and HSV-1 are the most common causes for viral encephalitis. Of note, the elderly population is susceptible to reactivation of latent varicella zoster virus (VZV), which results in shingles (Gilden et al., 2009). VZV is normally controlled by peripheral IFN response, but occasionally it can spread to CNS to cause encephalitis (Gilden et al., 2009; Kim et al., 2017). Fortunately, many people recover from these acute episodes; yet, a significant portion suffer from long term neurological sequelae (Swanson and McGavern, 2015; Klein et al., 2017). Rather than the irreversible damage caused by the pathogens, recent studies suggested a role of chronic inflammation that underlie neurological impairments, including conditions post WNV infection as mentioned earlier (Vasek et al., 2016). Therefore, prior exposure to CNS infections may have long-lasting neurocognitive consequences (Figure 2).

Although responsible for a common form of acute viral encephalitis, herpes simplex virus mostly carries out latent infection in the general population. Itzhaki et al. (1997) first detected HSV-1 DNA in the brain of 60% cases of apolipoprotein E gene (APOE-ε4) carriers and subsequently postulated the viral concept of AD: HSV-1 likely travels to the brain in middle age, where it remains in a latent state, and accumulation of damage from intermittent reactivation – direct viral action and major inflammatory effects – leads eventually to the development of AD (Itzhaki, 2018). Although the causal relationship between HSV-1 reactivation and AD is extremely difficult to prove in humans, the proposal is consistent with the concept of pathogenic chronic inflammation in CNS, as mentioned above. The IFN signaling, as a result of the cGAS-STING pathway activation in microglia, is indispensable for controlling HSV-1 infection in humans (Dupuis et al., 2003; Reinert et al., 2016). Therefore, IFN response required for resolving periodic HSV-1 activation may conceivably be a contributing factor in AD (Figure 2). Over the years, other viral pathogens have been detected in AD specimens and implicated in AD risk, among which noticeably are members of Herpesviridae. Increased human herpesvirus subtypes (HHV-6 and HHV-7) were independently identified from a multi-scale gene expression network analysis of late-onset AD (Readhead et al., 2018), though the study was later challenged (Jeong and Liu, 2019; Allnutt et al., 2020). On the other hand, while acute HSV-1 infection induced Aβ production in human induced neural stem cells (Cairns et al., 2020), Aβ was shown to play a protective role in brain against experimental infections by Herpesviridae, including HSV-1, and HHV-6 (Eimer et al., 2018). The interaction between Aβ and different types of microbes involves surface carbohydrate recognition, which rapidly seeds amyloid fibrils (Kumar et al., 2016; Eimer et al., 2018; Figure 1). In lieu of immune reaction, it remains to be shown if Aβ-entrapped viruses can be recognized by microglia and elicit effective IFN response.

Under pathological conditions, self-derived molecules can potently trigger inflammatory response in the absence of an infection. As discussed earlier, extracellular amyloid plaques with sequestered nucleic acids are recognized by microglia and elicit an antiviral response analogous to that during viral infection (Figure 1). Of note, sequence analysis of RNA isolated from AD neuritic plaques identified transcripts from cortical neurons, marking a self origin of plaque-associated nucleic acids (Ginsberg et al., 1999). Conceivably, such intrinsic IFN response would deter opportunistic viral infection or reactivation in AD brain, a point that awaits further investigation (Figure 1). On the other hand, dysregulated nucleic acid catabolism also results in aberrant IFN production. For example, mutations of deoxyribonuclease *TREX1* or ribonuclease *RNASEH2* lead to the accumulation of aberrant cytosolic nucleic acid species, IFN production, and encephalopathy in Aicardi-Goutieres syndrome (Rodero and Crow, 2016). Under neurodegenerative conditions, dead brain cells may release nucleic acids and other alarmin molecules thus stimulating an inflammation response. Although peripheral immune cells discern immunogenic vs. non-immunogenic cell death (Green et al., 2009), how microglia innately respond to different forms of CNS cell death is not known at this time. It is thus important to examine if additional endogenous agents stimulate innate IFN response in AD besides amyloid plaques.

It’s well known that age is the most important risk factor for late-onset AD (LOAD). In normal aging brain, heightened IFN signaling from microglia inside the parenchyma and choroid plexus, an epithelial tissue located within the ventricles, has been shown to be detrimental to neurogenesis and cognitive function (Baruch et al., 2014; Deczkowska et al., 2017). On the other hand, LOAD is a polygenic disease, where a number of risk polymorphism and rare variants exert their functions from microglia and/or involved in immunity (Zhang et al., 2013; Huang et al., 2017; Kunkle et al., 2019). The implication of ISGs as risk factor of AD (Salih et al., 2019) together with IFN upregulation in aging brain suggest that IFN pathway may have a profound influence on AD pathogenesis (Figure 2).

Adult Down syndrome (DS) patients, who mostly carry trisomy 21 in their genome, unanimously develop the neuropathological changes of AD (Lott and Head, 2019). Besides amyloid precursor protein gene, four of the six IFN receptors, IFNAR1, IFNAR2, IFNGR2, and IL10RB, are encoded in the extra chromosome 21, which results in profound peripheral IFN response and autoinflammation in DS patients (Kola and Hertzog, 1997; Sullivan et al., 2016, 2017). Interestingly, mice bearing trisomy 16, which contains many orthologs from human trisomy 21, benefited from receiving antibodies blocking type I IFN (IFNα/β) and type II IFN (IFNγ) at development stage *in vivo*, whereas blocking IFNγ signaling rescued the premature death of trisomy 16 cortical neurons *in vitro* (Maroun, 1995; Hallam and Maroun, 1998; Hallam et al., 2000). By contrast, IFNγ blockade did not affect microglial activation nor synapse loss in amyloid β model (Roy et al., 2020). Since both type I and type II IFNs activate an overlapping JAK/STAT pathway to convey antiviral protection (Liu et al., 2012), these findings imply DS as a disease likely more affected by interferon activation.

In summary, we have described the IFN pathway activation, a prototypic antiviral immune response, as a major component of CNS neuroinflammatory network in AD and connected IFN response to various endogenous, pathological, infectious and genetic risk factors that have been implicated in AD pathogenesis. The discussion here is largely focused on the molecular events originated within CNS, but peripheral IFN, manifested as a result of viral infection, autoimmune condition or drug administration, nevertheless affects brain functions (Blank et al., 2016). Therefore, many important questions remain to be investigated to advance our fundamental understanding of AD more in the future.

## Data Availability Statement

The original contributions presented in the study are included in the article/supplementary material, further inquiries can be directed to the corresponding author.

## Author Contributions

WC formulated the concepts and wrote the manuscript. ER contributed to the manuscript writing and figure preparation. All authors contributed to the article and approved the submitted version.

## Conflict of Interest

The authors declare that the research was conducted in the absence of any commercial or financial relationships that could be construed as a potential conflict of interest.
